# Automated plasmid design for marker-free genome editing in budding yeast

**DOI:** 10.1093/g3journal/jkae297

**Published:** 2024-12-17

**Authors:** Lazar Stojković, Vojislav Gligorovski, Mahsa Geramimanesh, Marco Labagnara, Sahand Jamal Rahi

**Affiliations:** Laboratory of the Physics of Biological Systems, Institute of Physics, École polytechnique fédérale de Lausanne (EPFL), CH-1015 Lausanne, Switzerland; Laboratory of the Physics of Biological Systems, Institute of Physics, École polytechnique fédérale de Lausanne (EPFL), CH-1015 Lausanne, Switzerland; Laboratory of the Physics of Biological Systems, Institute of Physics, École polytechnique fédérale de Lausanne (EPFL), CH-1015 Lausanne, Switzerland; Laboratory of the Physics of Biological Systems, Institute of Physics, École polytechnique fédérale de Lausanne (EPFL), CH-1015 Lausanne, Switzerland; Laboratory of the Physics of Biological Systems, Institute of Physics, École polytechnique fédérale de Lausanne (EPFL), CH-1015 Lausanne, Switzerland

**Keywords:** pop-in/pop-out, genomic editing, marker-free, scarless, budding yeast

## Abstract

Scarless genome editing in budding yeast with elimination of the selection marker has many advantages. Some markers such as *URA3* and *TRP1* can be recycled through counterselection. This permits seamless genome modification with pop-in/pop-out, in which a DNA construct first integrates in the genome and, subsequently, homologous regions recombine and excise undesired sequences. Popular approaches for creating such constructs use oligonucleotides and PCR. However, the use of oligonucleotides has many practical disadvantages. With the rapid reduction in price, synthesizing custom DNA sequences in specific plasmid backbones has become an appealing alternative. For designing plasmids for seamless pop-in/pop-out gene tagging or deletion, there are a number of factors to consider. To create only the shortest DNA sequences necessary, avoid errors in manual design, specify the amount of homology desired, and customize restriction sites, we created the computational tool PIPOline. Using it, we tested the ratios of homology that improve pop-out efficiency when targeting the genes *HTB2* or *WHI5*. We supply optimal pop-in/pop-out plasmid sequences for tagging or deleting almost all S288C budding yeast open reading frames. Finally, we demonstrate how the histone variant Htb2 marked with a red fluorescent protein can be used as a cell-cycle stage marker, alternative to superfolder GFP, reducing light toxicity. We expect PIPOline to streamline genome editing in budding yeast.

## Introduction

**Fig. 1.**
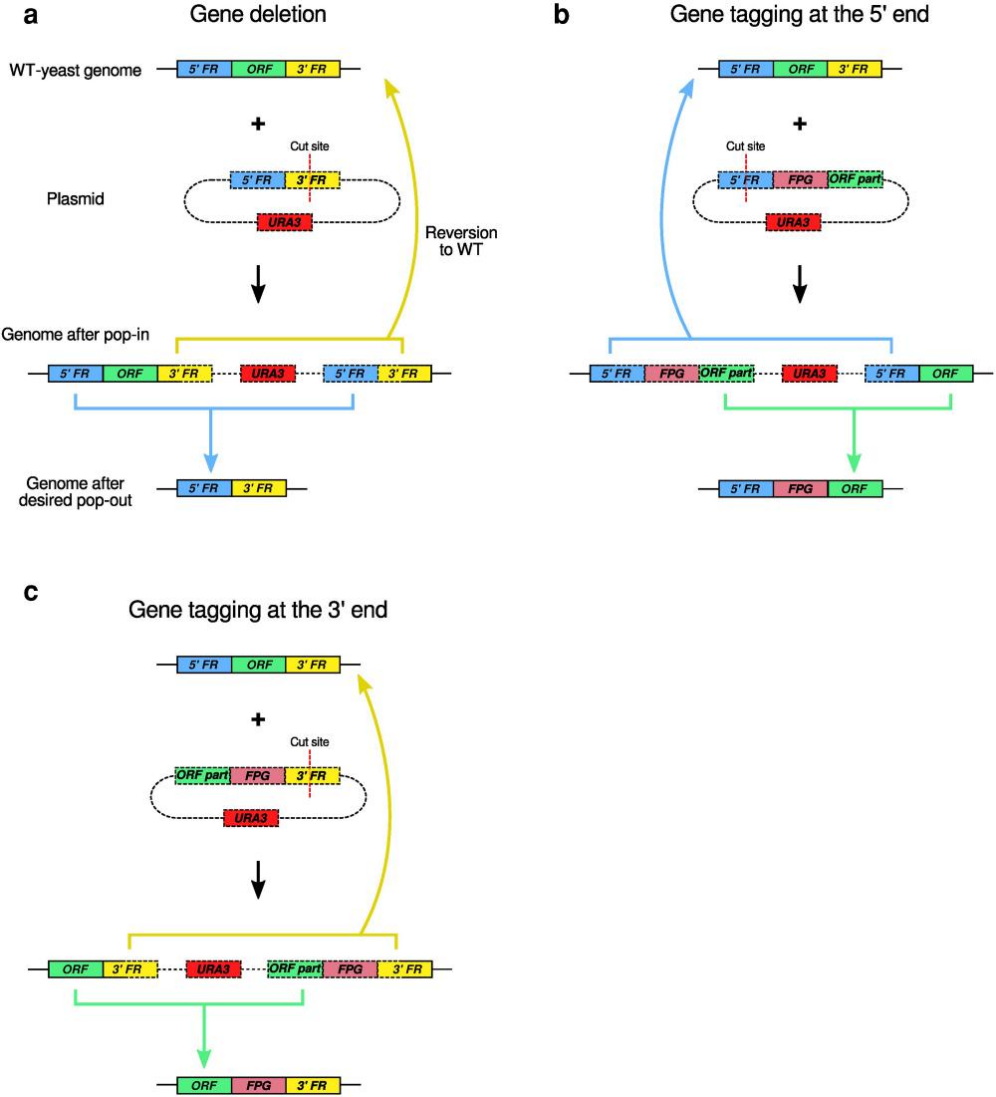
Gene editing using PIPO. a) Deletion of an ORF. Here, the pop-in homology comes from the 3′ FR and pop-out homology from the 5′ FR. In principle, the cut site for linearizing the plasmid could alternatively have been in the 5′ FR sequence and the pop-out homology could come from the 3′ FR (not shown). b) Genomic edit to tag the expressed protein at the N-terminus. In this illustration, the pop-in homology comes from the 5′ FR and the pop-out homology from the ORF. In principle, the cut site for linearizing the plasmid could alternatively have been in the partial ORF sequence and the pop-out homology could come from the 5′ FR (not shown). c) Genomic edit to tag the expressed protein at the C-terminus. Here, the pop-in homology comes from the 3′ FR and the pop-out homology from the ORF. The roles of the 3′ FR and ORF for pop-in and pop-out homology could have been reversed (not shown). a–c) PIPO is performed using a custom-designed plasmid that allows seamless 2-step genome editing. In the first step, the PIPO plasmid is integrated and cells are selected for the *URA3* marker. In the second step, cells are selected for the loss of *URA3*. Depending on the DNA sequences that recombine, pop-out can either lead to reversion to the WT yeast genome (curved arrows pointing up) or to the desired genome edit (colored arrows pointing down). FPG, fluorescent protein gene; ORF, open reading frame.

Budding yeast is an important model organism in genetics, molecular biology, and synthetic biology. Its genome is routinely modified in a highly targeted manner without specialized endonucleases; the homologous recombination machinery integrates the exogenous DNA into the yeast genome (Rothstein, 1991). Transformation can be performed with high efficiency but requires auxotrophic or antibiotic resistance markers to screen for transformed cells (Sikorski and Hieter 1989; Pronk 2002; Chee and Haase 2012). To reuse markers, researchers utilize a 2-step method, called pop-in/pop-out (PIPO), that removes the extraneous DNA after transformation, including the marker (Scherer and Davis 1979; Längle-Rouault and Jacobs 1995; Sadeghi *et al*. 2022). Thus, in principle, an arbitrary number of modifications can be introduced in the genome. The method can be applied for deletions of open reading frames (ORFs) or seamless tagging with fluorescent proteins, for example (Fig. 1).

PIPO involves initial positive selection followed by negative selection (counterselection). Both of these steps select for cells in which a recombination event occurs: in the pop-in step, the sequence of interest integrates into the yeast genome, thereby introducing the selectable marker. In the pop-out step, cells that have lost the marker are selected. Critically, depending on the recombination site, these cells may revert to the wild-type (WT) genome (curved arrows pointing up in Fig. 1), which is undesired, or result in the desired genome modification (colored arrows pointing down in Fig. 1).

**Fig. 2.**
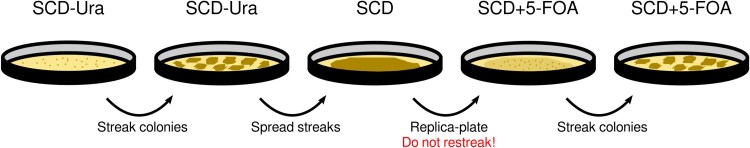
Experimental steps in PIPO. Cells transformed with the PIPO construct are selected on synthetic complete media plates lacking uracil (SCD-Ura). Transformant colonies are then restreaked on fresh SCD-Ura plates. One or multiple of these patches are then spread onto nonselective SCD plates (1 SCD plate for each original transformant colony). After 1 day, cells are transferred to an SCD plate containing 5-FOA (SCD + FOA) by replica plating. After about 4 days, single colonies from the SCD + 5-FOA plate are restreaked onto a fresh SCD + FOA plate.

Marker genes that can be both selected for and against in budding yeast are in the lysine (Chattoo *et al*. 1979), tryptophan (Toyn *et al*. 2000), or uracil (Boeke *et al*. 1987) biosynthesis pathways. Among them, the *URA3* gene is often used due to its short length (267 amino acids) and the low spontaneous reversion frequency of the genomic *ura3Δ* copy. *URA3* is selectable on uracil-deficient media and counterselectable on media containing 5-fluoroorotic acid (5-FOA) (Boeke *et al*. 1987). The experimental process is depicted in Fig. 2.

An advance in the design of cassettes for seamless genome modifications was to directly use PCR-generated DNA sequences to replace a genomic sequence of interest (Manivasakam *et al*. 1995; Oldenburg 1997), used, for example, in the creation of the yeast knockout collection (Winzeler *et al*. 1999). In doing so, researchers skip the plasmid construction step. This has led to the design of several innovative strategies, such as “delitto perfetto” (Storici and Resnick 2006) and 50:50 (Horecka and Davis 2014). However, PCR-based transformation is limited in the amount of homology that can be created for genomic integration. Commercially available oligonucleotides are typically shorter than 120 nucleotides. The efficiency of such transformations is dependent on the strain background (Manivasakam *et al*. 1995). In addition, oligonucleotides are less stable than plasmids and cannot be easily regenerated in typical biological laboratories. This led us to focus on plasmid-based PIPO, in part, since the prices for DNA synthesis and cloning have dropped sharply.

There are a number of parameters that have to be simultaneously optimized for plasmid-based PIPO, making manual design tedious:

- sufficient homology for plasmid integration (pop-in), where the minimum homology is specified by the user;
- specific amount of homology for plasmid pop-out;
- minimizing the length of the DNA sequence that needs to be synthesized;
- uniqueness of restriction site for plasmid integration; and
- restriction sites for subcloning different tags such as fluorescent protein genes.

These criteria can be formalized for a computational pipeline. Here, we present PIPOline (pop-in/pop-out pipeline), a Python-based algorithm for the design of plasmids for PIPO-based genome editing. Additionally, we use PIPOline to show how the probability of the desired pop-out can be fine-tuned through sequence length optimization. Finally, labeling the histone variant Htb2, we demonstrate how cell-cycle phases can be observed using the fast-folding red fluorescent protein ymScarletI, alternative to superfolder GFP (sfGFP).

## Methods

### Yeast strain construction

Transformations of budding yeast were performed using the high-efficiency LiAc/DNA carrier/PEG (polyethylene glycol) protocol (Gietz and Schiestl 2007). All strains are derivatives of W303 (*leu2-3,112 trp1-1 can1-100 ura3-1 ade2-1 his3-11,15*). The list of strains used is supplied in Supplementary Table 1.

### Media

We used synthetic complete media (“Synthetic complete (SC) medium,” 2016) (SC) with or without uracil (Ura) and supplemented with 2% (w/v) glucose (D) or 3% (w/v) galactose (G). For preparing solid SC plates, we used 2% agar. For experiments with 5-FOA (SCD + 5-FOA), we used the standard recipe with 0.1% 5-FOA (Boeke *et al*. 1987; “5-Fluoroorotic Acid (5-FOA) Plates,” 2016).

### Plasmid construction

Plasmids designed by PIPOline were assembled using restriction enzyme cloning and T4 ligase (NEB). The regions homologous to the yeast genome were amplified using high-fidelity Phusion polymerase (Thermo Fisher Scientific) with genomic DNA as a template. Yeast codon-optimized *mScarletI* (*ymScarletI*) was adapted from Botman *et al*. (2019) by removing cut sites of frequently used restriction enzymes and was synthesized by GenScript. Plasmids were propagated in XL10 *Escherichia coli* in LB medium supplemented with ampicillin. The list of plasmids used is supplied in Supplementary Table 2.

### Pop-in/pop-out

Cells were transformed with the pETURALEU plasmid containing the insert used to modify the locus of interest. After 3 days, isolated colonies were restreaked onto fresh SCD-Ura plates. Upon confirming that the plasmid integrated as expected into the yeast genome by PCR and selection on –Leu plates, we proceeded to the pop-out step. First, cells were grown on nonselective media (SCD) plates to enable the survival of the colonies in which the *URA3* marker was lost. To spread cells onto SCD plates, single-colony streaks from SCD-Ura were briefly resuspended in 100 µL of liquid SCD medium and then evenly spread onto agar plates. After 24 h, cells were replicated on SCD + 5-FOA plates to select for cells that did not contain the *URA3* marker. Cells were grown for 4 days on FOA after which single colonies were picked and regrown on SCD + 5-FOA plates before the screen. For a graphical summary, see Fig. 2.

### Fluorescence microscopy

Recordings were made using a Nikon Ti-2E microscope equipped with a 20× objective and a Hamamatsu Orca-Flash4.0 camera. For scoring the pop-out efficiency, the strains were grown in 96-well plates in synthetic complete medium. The strains were scored based on the intensity of the nuclear foci in the red fluorescence channel with 50 ms exposure time. For running time course experiments, cells were grown in CellAsic microfluidic chips and recorded using the same microscope with a 63× objective. For the timelapse recordings, images were taken every 5 min.

### Data analysis

For processing single-cell microscopy experiments, cells were segmented and tracked using YeaZ (Dietler *et al*. 2020), available at https://github.com/rahi-lab/YeaZ-GUI. For analyzing single-cell time courses, we used a custom-based code written in Python 3.11.3.

Individual cell cycles shown in Fig. 5 had variable duration. To make their dynamics comparable, we normalized the time axis so that timepoint 0 corresponds to mother–daughter anaphase and 1 to the budding of the daughter cell. To calculate the average time course, single-cell curves were interpolated at 30 timepoints and smoothed using spline interpolation (splrep function from scipy 1.10.1 Python package with smoothing factor 0.02).

## Results

### PIPOline: pipeline for the design of PIPO plasmids

We designed PIPOline for 2 kinds of genomic modification: ORF deletion or protein tagging at the N- or C-terminus. The inputs to the PIPOline program are listed in Table 1 (additional information in Supplementary Notes 1–6); the names of the corresponding command line parameters are supplied in Supplementary Table 1. PIPOline can in principle design a PIPO plasmid for any vector backbone. To easily eliminate *URA3* revertants during pop-in and pop-out steps, we created a dually marked backbone, pETURALEU, available from the Addgene plasmid repository. In addition to the *URA3* auxotrophic marker, the pETURALEU plasmid contains the *LEU2* gene. In a strain with a *LEU2* loss-of-function mutation, *URA3* revertants can be easily distinguished from cells having undergone successful pop-in or pop-out by additionally testing for leucine auxotrophy. In contrast to pETURALEU, the pLS406 shuttle vector (Scutteri *et al*. 2024) only has a *URA3* marker and was miniaturized and depleted of most restriction sites outside the multiple cloning site (MCS) to improve the efficiency of complex insert engineering such as for PIPO.

**Table 1.** Inputs to PIPOline program.

User-specified data	Sources and examples
Vector backbone sequence	We supply the sequence of the vector backbone pETURALEU containing *URA3* as well as secondary *LEU2* marker in Supplementary Note 3
Location of the MCS for inserting the synthetic DNA sequence in the vector backbone	Supplied in Supplementary Note 1 for pETURALEU
Minimum number of base pairs of homology for plasmid integration (pop-in) at the target locus on each side of the linearization cut site	As the default value, we use 70 bp as the minimum amount needed on each side of the cut site
Ratio of “good” vs “bad” homology for pop-out, that is, sequences that should recombine to lead to the desired genomic change vs sequences that would recombine to remove the whole plasmid, reverting to the original genome	We show in this work that a ratio of equal or greater than 2 is appropriate
Sequence of gene to be tagged or deleted, specifically 1,000 bp upstream of the ORF, ORF, and 1,000 nucleotides downstream of the ORF	Found on yeastgenome.org for budding yeast genes. We additionally supply all sequences for strain S288C in the Supplementary Files
List of restriction enzymes that can be used	Suggested list in Supplementary Note 4 can be customized
Linker to be used if tagging a gene	A default linker sequence is supplied in Supplementary Note 5
List of sequences of fluorescent protein genes which could be used for PIPO tagging and all of which the plasmid should be compatible with	Sequence of ymScarletI fluorescent protein gene supplied in Supplementary Note 6

**Fig. 3.**
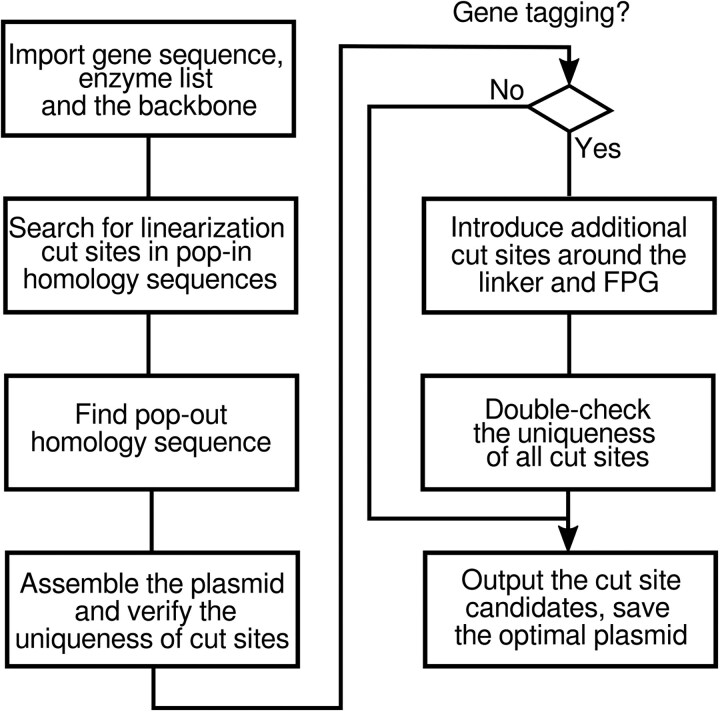
PIPOline process diagram. FPG, fluorescent protein gene.

The steps in PIPOline are enumerated below and depicted in Fig. 3. In summary, dependent on the genomic editing task with PIPOline, 2 sequences from different regions of a gene of interest (GOI) need to be included in the PIPO plasmid, taken from the 5′ flanking region (FR), ORF, or 3′ FR. The algorithm loops through candidate restriction sites in the 2 regions that may be used for linearizing the final plasmid. The pipeline evaluates whether the PIPO plasmid can be built based on the candidate restriction site according to a specific set of rules. If not, the program notifies the user about the reason why a candidate linearization cut site has to be disregarded. Otherwise, the algorithm outputs the sequence to be synthesized for each linearization cut site, which can be synthesized and cloned into the specified vector backbone:

1. For tagging or deleting ORFs, 2 regions of the GOI are used to create homology for pop-in and pop-out, respectively: (1) 5′ FR and ORF for tagging the ORF at the 5′ terminus, (2) 3′ FR and ORF for tagging the ORF at the 3′ end, or (3) 5′ FR and 3′ FR for deleting the ORF. To determine the 5′ FR, ORF, and 3′ FR, the PIPOline algorithm assumes that the user-supplied GOI sequence consists of 1,000 bp downstream of the ORF, followed by the ORF, and 1,000 bps upstream of the ORF. Parts of both regions are used in the PIPO plasmid, one for pop-in and the other for pop-out, as determined by the following steps. The code performs 2 checks: (1) for Start and Stop codons at the expected positions and (2) whether there is overlap with another ORF (based on similarity to overlapping ORFs in budding yeast strain S228C) and alerts the user. For each budding yeast gene, the required DNA sequence can be readily downloaded from the yeastgenome.org database (Wong *et al*. 2023). We additionally supply all such sequences for budding yeast strain S288C in the Supplementary Files.
2. One of the two regions will be used for integrating the plasmid into the genome (“pop-in homology,” boxes containing cut sites in Fig. 1). A sequence from this region will be included in the PIPO plasmid and contains the cut site used to linearize the plasmid with restriction enzymes prior to transformation. To identify the linearization cut site and the pop-in homology, PIPOline searches in both regions for unique restriction sites corresponding to the user's preferred restriction enzymes (Table 1). For each candidate linearization cut site, PIPOline measures the size of the region on both sides of the cut site. These sequences must be longer than a user-specified minimum. If cutting the plasmid at the candidate cut site results in less than the specified minimum homology, the linearization cut site is not considered further by the program. For example, the cut site in Fig. 1a has to be sufficiently far from the 5′ FR sequence to its left. As pop-in homology, PIPOline takes the whole sequence between the cut site and the neighboring region as well as a sequence of length corresponding to the minimum homology on the other side of the cut site.
3. The region which is not used for linearizing the plasmid and integration is required for the counterselection step (“pop-out homology,” e.g. 5′ FR blue box in Fig. 1a). The user-supplied parameter *R*_homo_ specifies the ratio of pop-out to pop-in homology. PIPOline checks whether a sufficiently long sequence is available in the second region (i.e. region not used for pop-in homology) and uses it for pop-out homology. If a sufficiently long sequence is not available, the maximal available sequence is taken for pop-out homology, and a warning message is associated with this linearization cut site and construct.
4. The sequences of pop-in and pop-out homology as well as a linker and fluorescent-protein gene (FPG) for tagging the GOI are assembled for a candidate PIPO construct.
5. Unique restriction sites for cloning the insert into the MCS of the vector backbone, maintaining the uniqueness of the linearization restriction site, are identified. If this is not possible, the linearization cut site is disregarded and a message describing the error is displayed.
6. For ORF tagging, PIPOline searches for additional cut sites to be added around the linker and around the fluorescent protein gene sequence. This allows for straightforward exchange of these pieces in the future. The program uses a user-defined list of restriction enzymes with 6 bp long cut sites and verifies that they do not introduce a stop codon.
7. PIPOline outputs the list of all linearization cut site candidates. Cut sites which cannot be used for constructing a PIPO plasmid respecting the above rules are listed as well, together with the reason for discarding them. If a linearization cut site can be used for a PIPO plasmid, the program outputs the sequences that need to be synthesized, the restriction sites for subcloning, and the total length of the insert DNA. The linearization cut site that meets all criteria and requires the minimal amount of DNA to be synthesized is considered to be optimal, and the corresponding assembled plasmid sequence is saved as a FASTA file.

We tested the algorithm with all 6,039 budding yeast strain S288C genes identified at yeastgenome.org for ORF tagging or deletion with *R*_homo_ = 2 (see below). PIPOline could find sequences satisfying the above criteria for 99.42% of the plasmids (18,012 out of 18,117) needed to tag or delete each gene. Further statistics regarding the plasmids are supplied in Supplementary Figs. 1 and 2. In the Supplementary Files, we supply all plasmid sequences for deletion or tagging; the desired tag sequence, with potential duplicate restriction sites removed by switching synonymous codons, has to be inserted by the user.

PIPOline is available as a command line tool (Data availability) and has been tested on Linux Ubuntu 21.04, Windows 10, and Windows 11 operating systems. Examples of calls for different genome editing tasks are given in Supplementary Note 2. The tool can also be run in an online graphical user interface available at https://colab.research.google.com/drive/12hle7b94_j6DbAeaJFaCH6jbU1yM1f_Z.

### Testing PIPOline and maximizing successful pop-outs by tuning *R*_homo_

In the pop-out step, the pop-in and pop-out homology of the integrated PIPO plasmid can recombine with homologous sequences in the genome, resulting either in the desired genomic change or reversion to the WT sequence (colored arrows in Fig. 1). Therefore, the colonies that emerge after counterselection have to be screened for the desired pop-out. We investigated whether tuning the ratio of lengths of pop-in and pop-out homology could favor one recombination event over the other. This would guide plasmid design for efficient pop-out and reduce the manual screening burden. Such a strategy has been considered before (Rothstein 1991), has been validated in fission yeast (Gao *et al*. 2014), and would plausibly work in budding yeast due to the dependence of recombination frequencies on the lengths of homologous sequences (Ahn *et al*. 1988; Yuan and Keil 1990; Sugawara and Haber 1992; Jinks-Robertson *et al*. 1993).

To test the idea that competition between the 2 spontaneous recombination outcomes is decided by the ratio of lengths of homologous sequences, we used PIPOline to construct a series of plasmids that would C-terminally tag Htb2 or Whi5. In each series, the pop-in homology sequence was kept constant, while the pop-out homology length was varied. Thus, *R*_homo_ ranged between 0.5 and 4 for Whi5 tagging and between 0.5 and 2 for Htb2 tagging. (Extending *R*_homo_ to 4 for Htb2 tagging was not possible since it would have led to a nonunique linearization cut site.)

We created the PIPOline inserts with the different *R*_homo_ values in the pETURALEU plasmid backbone. (Alternatively, these inserts could be obtained from commercial gene synthesis vendors, cloned in the desired vector backbone.) We linearized plasmids with the enzymes chosen by PIPOline and performed a standard LiAc chemical transformation with 0.5 µg of DNA. Transformants were selected on synthetic complete glucose agar plates without uracil (SCD-Ura) (Fig. 2). Starting with 5 mL of exponentially growing culture, we typically obtain up to 30 transformants. Colonies were restreaked as patches onto fresh SCD-Ura plates to minimize the chance that untransformed cells could be transferred to the next steps. To allow spontaneous recombination events to pop out the undesired sequences, we then streaked out 2–3 of the patches onto nonselective growth medium plates. For the next step, we found it important to replica plate from the nonselective growth medium plate onto a 5-FOA plate and not streak a sample from the nonselective medium plate (Methods). We hypothesize the reason to be that many genomic edits result in a fitness penalty (Breslow *et al*. 2008; Fudala and Korona 2009), which would increase the relative number of cells with revertant pop-outs vs desirable pop-outs if cells are mixed (e.g. restreaked). Replica plating from nonselective to counterselective plates should not change the relative number of colonies.

When pop-out occurs in the desired direction, the *URA3* and *LEU2* markers are removed from the genome, leaving the fluorescent protein at the ORF 3′ terminus. On the other hand, if the whole plasmid pops out from the genome, the gene remains unlabeled. We scored the type of resulting pop-out using 2 different strategies: fluorescence microscopy for *HTB2* tagging and PCR for *WHI5* tagging.

**Fig. 4.**
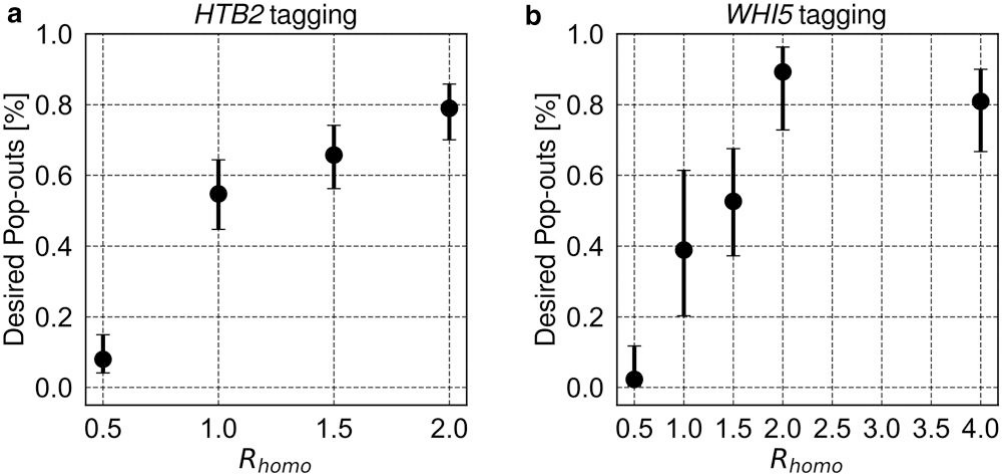
Increasing the ratio of lengths of pop-out vs pop-in homology (*R*_homo_) leads to a higher probability of the desired pop-out when tagging either a) *HTB2* or b) *WHI5* in budding yeast. Error bars show 95% confidence intervals calculated using the Wilson score method. The numbers of analyzed individual colonies are shown in Supplementary Table 3.

As expected, the probability of the desired pop-out for equal lengths of pop-in and pop-out homology sequences (*R*_homo_ = 1) was close to half (55% for *HTB2* and 39% for *WHI5*) (Fig. 4; Supplementary Table 3). Interestingly, for both *HTB2* and *WHI5*, we observed that the efficiency of the desired pop-out was tunable across a range of probabilities by modulating *R*_homo_. Reducing *R*_homo_ to 0.5 made the desired pop-outs very improbable (8% for *HTB2* and 2% for *WHI5*). On the other hand, increasing it to 2 led to 79 or 89% of the colonies having the desired pop-out, when tagging *HTB2* or *WHI5*, respectively (Fig. 4). Increasing *R*_homo_ above 2 in the case of *WHI5* had no apparent effect on pop-out efficiency.

**Fig. 5.**
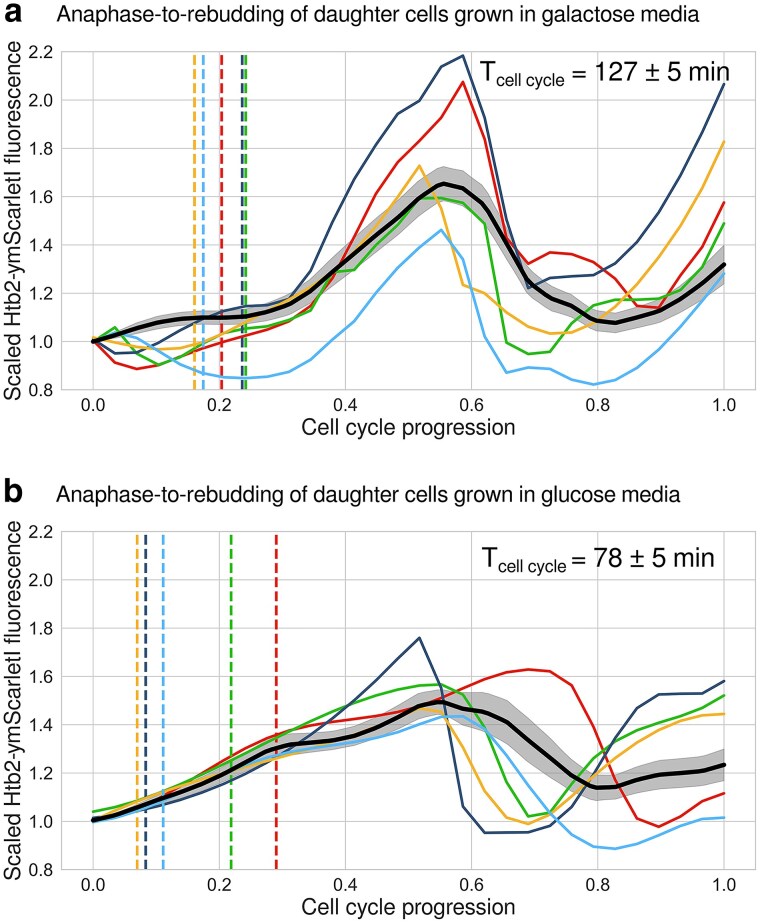
Htb2-ymScarletI dynamics in single daughter cells, from mother–daughter anaphase at time 0 to daughter rebudding at time 1. Cells were grown in synthetic complete media supplemented with a) 3% w/v galactose or b) 2% w/v glucose. Colored traces illustrate single-cell time courses while population averages are denoted in black. Shown are total fluorescent intensities, scaled by their first timepoint. To accommodate different cell-cycle lengths, as in a previous study involving histone synthesis tracking (Guerra *et al*. 2022), we normalized the time axis. Dashed lines show budding events, which in slowly cycling cells a) precede histone accumulation. In media supplemented with galactose a), the average budding-to-rebudding time was 127 ± 5 min (mean ± standard error of the mean), while in media with glucose b), it was 78 ± 5 min. Activation of histone synthesis in S-phase can be observed in the slow cell cycles a) but not in the fast ones b). The numbers of analyzed cells were a) 29 and b) 13. The standard errors of the mean (shaded area around the thick black line in both of the panels) showed that this number of cycles was sufficient to estimate the dynamics precisely.

The pop-out efficiency was scored using at least 2 different transformed (pop-in) colonies. We observed that in some cases, some colonies transformed with the PIPO plasmid never popped out the plasmid in the desired manner despite a relatively large *R*_homo_ (1.5 and 4). We did not identify the reason for this, but similar observations were made for a fission yeast pop-out plasmid and were explained by repair of the plasmid DNA with chromosomal sequences (Gao *et al*. 2014). We exclude such colonies from the statistics presented in Fig. 4 based on the very different pop-out efficiency compared to other colonies transformed with the same plasmid. This observation implies that it is best to use *R*_homo_ of about 2 or larger and also use more than one transformant when counterselecting for pop-out (Discussion).

Based on these results, we applied PIPOline to tagging 7 genes and deleting 8 genes. We ordered the insert corresponding to the PIPOline output to be synthesized and cloned into the pETURALEU vector backbone. While we did not quantify the number of pop-out colonies screened, we readily succeeded in tagging *CDC5*, *CLB2*, *CLB5*, *DNL4*, *NEJ1*, *SIC1*, and *SPC42* and in deleting *CLB3*, *CLB4*, *CLB5*, *CLB6*, *HEM14*, *HMX1*, *MRE11*, and *YMR315W*. The specific inserts and plasmid sequences we used are supplied in the Supplementary Files.

### Resolving cell-cycle phases using ymScarletI-marked histones

Finally, we wondered whether an Htb2-ymScarletI fusion created using PIPOline allows monitoring of histone accumulation in S-phase. Researchers have previously shown that the pulse-like pattern of histone transcription during S-phase (Gunjan *et al*. 2005; Eriksson *et al*. 2012) can be tracked using sfGFP (Garmendia-Torres *et al*. 2018; Guerra *et al*. 2022) but not with the relatively slow-folding mCherry (Garmendia-Torres *et al*. 2018). However, monitoring cellular processes using red fluorescent proteins produces less phototoxicity than with GFP. This is particularly important when frequent imaging is needed as in the case of dynamic cell cycle-related events.

The maturation time of ymScarletI in budding yeast is 12 min (Gligorovski *et al*. 2023), which is relatively close to that of sfGFP [7 min (Guerra *et al*. 2022)] compared to mCherry [52 min (Guerra *et al*. 2022)]. To test whether ymScarletI suffices to observe histone accumulation in single cells, we performed timelapse fluorescence microscopy. To correlate fluorescence levels more easily with the cell-cycle stage, we scaled the time axis: we assigned the moment in which the nuclei split into mother and daughter cells (anaphase) to timepoint zero and followed the daughter cell through budding until rebudding, assigned to timepoint 1.

Histone synthesis occurs in S-phase after cells have passed Start and committed to budding (Moll and Wintersberger 1976). Hence, we expected to see accumulating fluorescence around the time of budding (dashed lines in Fig. 5a). Indeed, shortly after budding, fluorescence levels started to rise and in the subsequent anaphase dropped to about half of the maximal value. Note that in the black curve which represents the average of the normalized single-cell time courses in Fig. 5, the peak is smeared out due to variability in the lengths of individual cell cycles. The region with flat fluorescence corresponded to G1 phase, which is substantially longer in daughter cells compared to mother cells (compare single-cell traces around timepoints 0 and 0.7). These results illustrate how 2 critical points of the cell cycle, G1-to-S transition and anaphase, can be visualized by a single red fluorescent marker.

In this and previously published experiments involving Htb2-sfGFP, cells were grown in galactose media, glucose-limited media (Garmendia-Torres *et al*. 2018), or minimal media (Guerra *et al*. 2022), resulting in a long cell-cycle period (127 min in our experiment and 145 min in Garmendia-Torres *et al*. (2018)). However, cells are often instead grown in rich media supplemented with 2% glucose, where the average cell cycle period is about 80 min (Rahi *et al*. 2016). Under such conditions, the transition from G1 to S-phase might not be as obvious. Indeed, for cells grown in glucose-rich media (Fig. 5b), the G1 period was less clear with fluorescence increasing more gradually. The time between anaphase and daughter budding was about 32 ± 3 min (mean ± standard error of the mean). Hence, ymScarlet may mature too slowly to observe histone dynamics in budding yeast cells growing in glucose-rich medium.

In summary, we conclude that *HTB2-ymScarletI* maturation allows visualization of progression through Start and anaphase in relatively slow cell cycles in a manner comparable to *HTB2-sfGFP*, the only other such marker available so far.

## Discussion

We present a pipeline for the automated design of plasmids for PIPO for tagging or deleting genes in budding yeast. PIPOline is customizable through user-defined parameters. It outputs a variety of possible strategies for a desired genomic edit and identifies the smallest insert that needs to be synthesized. PIPOline assumes the first and last 1,000 bp in the GOI file are 5′ FR and 3′ FR. Hence, designing plasmids for intergenic labeling using PIPOline is possible by adjusting the input file.

We experimentally mapped the probability of the desired recombination event depending on the ratio of pop-out and pop-in homology sequence lengths. This showed that having the pop-out homology twice as long as the pop-in homology leads to a large portion (>79%) of the popped-out colonies having the desired genomic edit. However, some colonies transformed with the PIPO plasmid did not yield any desirable pop-outs. Hence, the strategy that we propose is to use an *R*_homo_ of at least 2 and pop-out from at least 2 different transformed colonies. In our tests, this sufficed for finding at least 1 desired genotype.

PIPOline is well suited for genomic modifications in an era where commercial vendors offer gene synthesis and cloning into custom vectors at prices that are relatively affordable for typical laboratories. Using, for example, the doubly *URA3*- and *LEU2*-marked pETURALEU plasmid, only the minimal PIPOline-designed insert needs to be synthesized and cloned into the vector backbone. There are a number of advantages over oligonucleotide-based PIPO approaches: plasmids are easier to reproduce, reuse, share, and store long term. Because commercial oligonucleotides are limited in length to about 120 nt, the amount of homology that they can encode is limited to about 50 bp for insertion and 50 bp for pop-out, near the minimum limit for the homologous recombination machinery (Ahn *et al*. 1988; Yuan and Keil 1990; Sugawara and Haber 1992; Jinks-Robertson *et al*. 1993). Furthermore, the PCR step to create the PIPO construct becomes unreliable with long oligonucleotides. Finally, in the market relevant to our laboratory, the price per base pair for gene synthesis is lower than per nucleotide for oligonucleotides.

There are several similarities and differences between PIPO and CRISPR–Cas9-based scarless editing. Here, we assume that all of the cloning steps for CRISPR–Cas9 editing are performed by commercial gene synthesis service providers as well, eliminating differences in the amount of labor involved in preparing DNA constructs. Cas9 needs a single guide RNA (sgRNA) to target a specific genomic locus. The design of the sgRNA must respect the need for a protospacer-adjacent motif (PAM) and minimize off-target cleavage (Novarina *et al*. 2022), making it common to use computational tools for this step. After inducing a cut at the target site, a cotransformed template DNA is used by the cell's DNA repair machinery to introduce the desired genomic change (Novarina *et al*. 2022). The DNA constructs should be designed in such a way that in this step, the sequences recognized by Cas9 are eliminated to prevent recutting. Finally, the plasmids that have been introduced are counterselected or lost during proliferation in nonselective growth media. The number of steps and time needed for the 2 methods is roughly comparable, one transformation and one counterselection. Instead, we see the main difference in the troubleshooting that may be needed: the first step in PIPO, transformation with an integrating plasmid, is generally efficient and is performed routinely in yeast laboratories. Failure modes and countermeasures are well known. The factors important for the second step in PIPO, pop-out by spontaneous recombination, have been characterized in our work presented here. On the other hand, what steps to take when CRISPR–Cas9 genome editing fails is much less well known in typical yeast laboratories. This difference in the prevalence of expertise in the 2 techniques will diminish if CRISPR technology is more broadly adopted in yeast laboratories. Regardless, PIPOline fills a gap in making the currently most prevalent strategy for genome editing in budding yeast, which does not rely on endonucleases, more efficient. In addition, PIPOline is particularly important for tasks where PIPO has unique advantages such as genetic screens involving large allele replacements (Gao *et al*. 2014; Storey *et al*. 2019).

## Data Availability

pETURALEU is deposited with Addgene (ID: 223207). Supplementary Notes 1–6 present additional information regarding inputs to PIPOline. The Supplementary File contains all data as indicated in the manuscript. Supplementary items can be viewed at GSA FigShare: https://doi.org/10.25387/g3.27682254. The PIPOline code, tested in Python 3.11.3, is distributed under the MIT license and is available at https://github.com/rahi-lab/PIPOline. A web application is also available at https://colab.research.google.com/drive/12hle7b94_j6DbAeaJFaCH6jbU1yM1f_Z.
